# Induction of HIV Neutralizing Antibodies against the MPER of the HIV Envelope Protein by HA/gp41 Chimeric Protein-Based DNA and VLP Vaccines

**DOI:** 10.1371/journal.pone.0014813

**Published:** 2011-05-19

**Authors:** Ling Ye, Zhiyuan Wen, Ke Dong, Xi Wang, Zhigao Bu, Huizhong Zhang, Richard W. Compans, Chinglai Yang

**Affiliations:** 1 Department of Microbiology and Immunology and Emory Vaccine Center, Emory University School of Medicine, Emory University, Atlanta, Georgia, United States of America; 2 Agriculture Ministry Key Laboratory of Veterinary Public Health, Harbin Veterinary Research Institute, Harbin, People's Republic of China; 3 Central Laboratory, Tangdu Hospital, The Fourth Military Medical University, Xi'an, People's Republic of China; The University of Chicago, United States of America

## Abstract

Several conserved neutralizing epitopes have been identified in the HIV Env protein and among these, the MPER of gp41 has received great attention and is widely recognized as a promising target. However, little success has been achieved in eliciting MPER-specific HIV neutralizing antibodies by a number of different vaccine strategies. We investigated the ability of HA/gp41 chimeric protein-based vaccines, which were designed to enhance the exposure of the MPER in its native conformation, to induce MPER-specific HIV neutralizing antibodies. In characterization of the HA/gp41 chimeric protein, we found that by mutating an unpaired Cys residue (Cys-14) in its HA1 subunit to a Ser residue, the modified chimeric protein HA-C14S/gp41 showed increased reactivity to a conformation-sensitive monoclonal antibody against HA and formed more stable trimers in VLPs. On the other hand, HA-C14S/gp41 and HA/gp41 chimeric proteins expressed on the cell surfaces exhibited similar reactivity to monoclonal antibodies 2F5 and 4E10. Immunization of guinea pigs using the HA-C14S/gp41 DNA or VLP vaccines induced antibodies against the HIV gp41 as well as to a peptide corresponding to a segment of MPER at higher levels than immunization by standard HIV VLPs. Further, sera from vaccinated guinea pigs were found to exhibit HIV neutralizing activities. Moreover, sera from guinea pigs vaccinated by HA-C14S/gp41 DNA and VLP vaccines but not the standard HIV VLPs, were found to neutralize HIV pseudovirions containing a SIV-4E10 chimeric Env protein. The virus neutralization could be blocked by a MPER-specific peptide, thus demonstrating induction of MPER-specific HIV neutralizing antibodies by this novel vaccine strategy. These results show that induction of MPER-specific HIV neutralizing antibodies can be achieved through a rationally designed vaccine strategy.

## Introduction

It has been over 25 years since the identification of the human immunodeficiency virus (HIV) as the causative agent of AIDS [1], [2]. However, the tremendous research effort has not yet yielded an effective AIDS vaccine strategy. Earlier clinical trials using HIV Env-based subunit vaccines elicited antibodies that reacted with gp120 but were not neutralizing antibodies (NAbs), and vaccination failed to show protection against HIV infection [3]–[8]. The failure of these trials promoted a shift to the development of HIV vaccines that focus on eliciting T cell responses [9]–[11]. However, the disappointing outcome from a recent clinical trial of a T-cell-based vaccine regimen, the STEP trial conducted by Merck and HIV Vaccine Trials Network (HVTN), dealt another setback to AIDS vaccine development [12]. The failure of the STEP trial further reinforced the notion that an effective AIDS vaccine will need to induce both strong CTLs and broadly neutralizing antibodies (bNAbs) against HIV infection [13]–[15]. Nevertheless, effort to engineer vaccines that can induce HIV bNAbs has encountered great difficulties. Extensive sequence variation of concurrently circulating HIV strains poses a great challenge for inducing HIV bNAbs [16]. While conserved neutralizing epitopes have been identified in the HIV Env protein, induction of HIV bNAbs against such epitopes has been difficult largely due to camouflage of these cryptic sites by the highly variable sequences in the HIV Env surface subunit gp120 [17]. This is supported by structural studies of the HIV Env [18]–[20], which indicate that conserved neutralizing epitopes are either hidden behind variable loops or obscured by carbohydrates. Therefore, induction of HIV bNAbs will require the design and development of novel vaccine strategies that can overcome these obstacles.

The HIV Env transmembrane subunit gp41 serves to anchor the Env protein to cellular and viral membranes and mediate membrane fusion during virus entry into the cell. Although most of gp41 appears to be occluded in the HIV Env, a number of studies indicate that the membrane proximal external region of gp41 (MPER) is accessible to several HIV bNAbs and can be a promising target for vaccine design [21]. Several monoclonal antibodies (MAbs), which neutralize a broad range of primary HIV-1 isolates, are known to bind to adjacent epitopes located in the MPER [22]–[24]. The MPER is highly conserved and plays important roles in HIV Env incorporation and virus entry into the cells [25], [26], and the identification of these conserved neutralizing epitopes in the MPER spurred great effort to design vaccines for inducing HIV bNAbs against this region [27]. However, little success has been achieved by various approaches [28]–[33]. We previously reported the construction of an HA/gp41 chimeric protein, in which the gp120 subunit of HIV Env is replaced by the HA1 subunit of the influenza virus A/Aichi/2/68 (H3N2) HA protein [34]. This chimeric protein is efficiently transported to the cell surface and exhibits enhanced reactivity to monoclonal antibodies 2F5 and 4E10. In this study, we evaluated the immunogenicity of HA/gp41-based DNA and virus-like particle (VLP) vaccines in guinea pigs and investigated their ability to present the conserved epitopes in MPER for inducing MPER-specific HIV bNAbs.

## Results

### An unpaired Cys residue in HA/gp41 (Cys-14) affects folding of the chimeric protein

**Figure 1:**
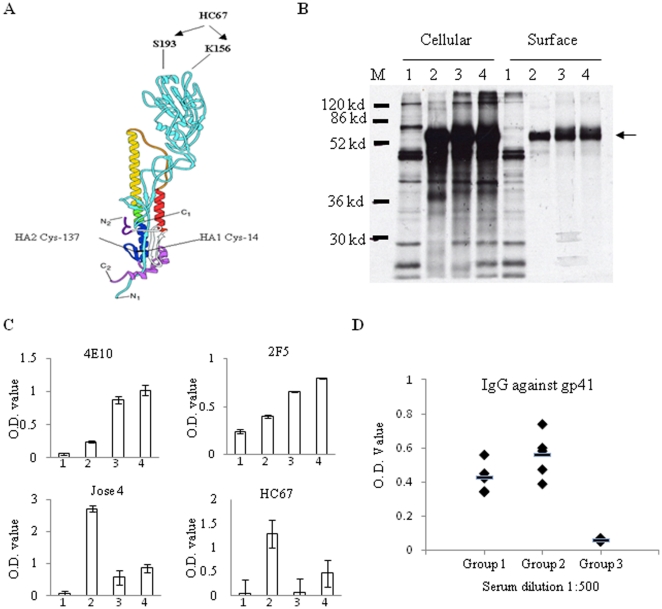
Characterization of the HA/gp41 and HA-C14S/gp41 chimeric proteins. **A.** Schematic diagram of the structure for influenza HA. Shown in the diagram are the positions of the Cys 14 of HA1 and Cys137 of HA2, which form a disulfide bond in the HA protein, and the position of the epitope (Ser 193 and Lys 156) recognized by the conformational antibody HC67 against HA. N1, C1, N2, and C2 represent N- and C-terminals of the HA1 and HA2 subunits respectively. **B.** Intracellular and surface expression of the HA/gp41 and HA-C14S/gp41 chimeric proteins. Protein expression was carried out using the recombinant vaccinia virus T7 expression system and detected by radioactive labeling coupled with surface biotinylation and immunoprecipitation using antibodies against HA as described in Materials and Methods. Lanes M, molecular weight marker; lanes 1, mock transfection by a plasmid DNA vector; lanes 2, influenza HA; lanes 3, HA/gp41; lanes 4, HA-C14S/gp41. Arrow indicates the position of the HA, HA/gp41, as well as HA-C14S/gp41 proteins in the SDS-PAGE. **C.** Comparison of antibody binding to HA, HA/gp41, and HA-C14S/gp41 by cell surface ELISA. Protein expression was carried out in a 96-well plate in triplicates as described above. At 16 hr post transfection, cells were fixed and then incubated with different antibodies at room temperature for 2 hr, including human MAbs 4E10 and 2F5 against gp41, rabbit polyclonal antibody Jose4 against HA, and a conformation-sensitive mouse MAb HC67 against HA as indicated. After incubation with primary antibodies, HRP-conjugated Goat-anti-human, rabbit, or mouse secondary antibodies were added to detect the amounts of the indicated antibody that bound to the cell surfaces, which were expressed by the average O.D. value from the wells expressing each protein and binding to each primary antibody respectively. 1, mock transfection by plasmid DNA vector; 2, influenza HA; 3, HA/gp41; 4, HA-C14S/gp41. **D.** Induction of gp41-specific antibodies by HA/gp41 and HA-C14S/gp41 DNA vaccines in mice. Mice (groups of 6) were immunized by HA/gp41 and HA-C14S/gp41 DNA vaccines twice at 4-week intervals, and blood samples were collected at 2 weeks after the second immunization for analysis of antibody responses against gp41 by ELISA. The levels of antibodies against gp41 in sera from individual mice in each group were expressed by O.D. values at 1∶500 serum dilution. Group1, immunization with HA/gp41 DNA vaccine; Group 2, immunization with HA-C14S/gp41 DNA vaccine, Group 3, immunization with plasmid DNA vector pCAGGS.

The HA/gp41 chimeric protein was originally constructed by fusing the HIV 89.6 Env gp41 to the C-terminus of the influenza virus A/Aichi/2/68 (H3N2) HA1 subunit [34]. As a result, the HA/gp41 chimeric protein contains an unpaired Cys residue, Cys-14, at the N-terminal end of the HA1 subunit, which in the wild type HA would form a disulfide bond with a Cys residue in the HA2 subunit (Cys-137) as shown in Figure 1A. To investigate whether this unpaired Cys-14 residue might affect the folding of the HA/gp41 chimeric protein, it was changed into a Ser residue by site-directed mutagenesis (amino acid sequence of this chimeric protein is provided in Figure S1). The resulting chimeric protein, designated as HA-C14S/gp41, was characterized for expression and transport to the cell surface in comparison with HA/gp41. As shown in Figure 1B, both the HA/gp41 and HA-C14S/gp41 chimeric proteins were expressed at comparable levels in the cells and were efficiently transported to the cell surface similar to the influenza HA, indicating that mutation of the Cys-14 residue did not affect the expression and transport of the chimeric protein. We next examined whether the mutation of the Cys-14 residue affected the reactivity of the chimeric proteins expressed on the cell surface to MAbs 2F5 and 4E10 by a cell surface ELISA. In addition, to determine whether the mutation of the Cys-14 residue may affect folding of the chimeric protein, we also included a MAb (HC67) that is conformation-sensitive and only binds to influenza HA under native and neutral pH conditions [35]. Further, a polyclonal rabbit-anti-HA antibody (Jose4) was also included to compare the level of protein expression on the cell surface. As shown in Figure 1C, MAbs 2F5 and 4E10 as well as to the polyclonal antibody Jose 4 bound to both HA/gp41 and HA-C14S/gp41 chimeric proteins expressed on cell surfaces at similar levels. However, whereas HC67 bound efficiently to the HA-C14S/gp41 chimeric protein, this conformation-sensitive MAb exhibited no significant reactivity with the HA/gp41 chimeric protein. These results indicate that folding of the HA1 subunit of the HA-C14/gp41 chimeric protein resembles more closely to the wild type HA compared to the HA/gp41 chimeric protein. We further carried out DNA immunization in mice to compare their immunogenicities for inducing antibodies against gp41. As shown in Figure 1D, although the differences are not statistically significant (p>0.05), immunization with the HA-C14S/gp41 DNA induced higher levels of antibody responses against gp41 on average than those induced by the HA/gp41 DNA.

### The HA-C14S/gp41 chimeric protein is incorporated into VLPs and forms more stable trimers compared to the HA/gp41 chimeric protein

**Figure 2:**
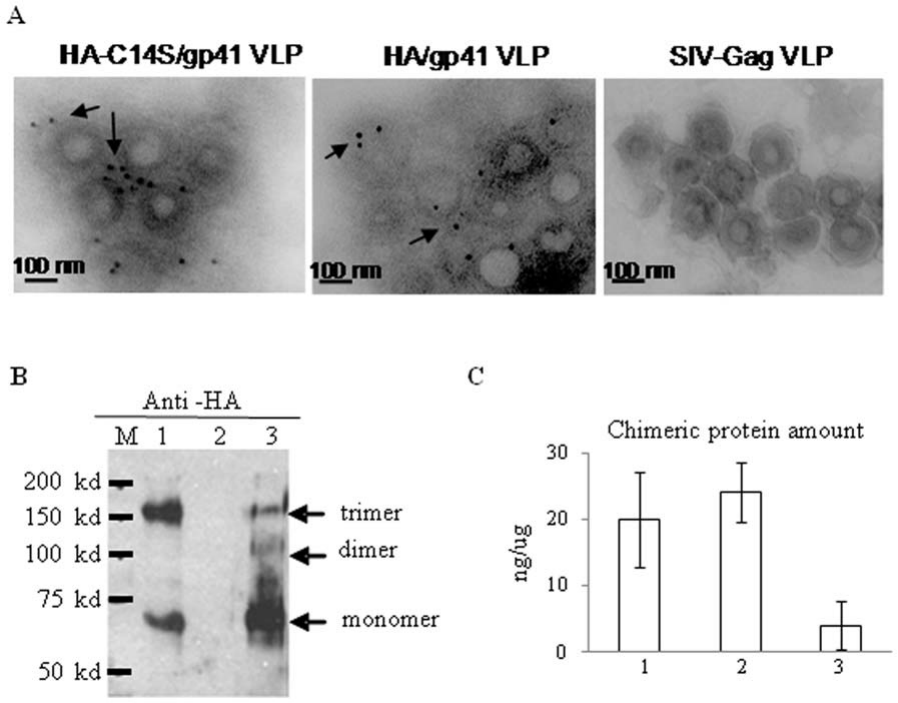
Characterization of the HA/gp41 and HA-C14S/gp41 VLPs. VLPs were produced in Sf9 insect cells using the recombinant baculovirus expression system and purified through a sucrose gradient as described in Materials and Methods. **A.** Characterization of VLPs by immune-gold-particle labeling and electron microscopy. Purified HA/gp41, HA-C14S/gp41, as well as the control SIV-Gag VLPs were coated onto a sample grid and then incubated with rabbit-anti-HA antibody Jose4 followed by incubation with gold-particle-conjugated goat-anti-rabbit secondary antibodies. The samples were then fixed with 1% uranyl acetate and then examined under an electron microscope. Arrows indicate gold-particle-labeled glycoproteins on the surface of VLPs. **B.** Detection of HA/gp41 and HA-C14S/gp41 chimeric protein oligomers in VLPs by Western blot. VLPs were lysed with 1% Triton X-100 and then mixed with non-reducing protein sample buffer followed by analysis by SDS-PAGE coupled with Western blot using antibodies against HA (Jose4). Lane M, molecular weight marker; 1, HA-C14S/gp41 VLP, 2, control SIV Gag VLP; 3, HA/gp41 VLP. **C.** Determining the amount of HA/gp41 and HA-C14S/gp41 chimeric proteins in VLPs by ELISA. VLPs were lysed with 1% Triton X-100 and then coated onto a microtiter plate at 1 µg per well, and the amount of chimeric proteins were detected by ELISA using rabbit-anti-HA antibody Jose4. Control wells were coated with 1 µg of SIV-Gag VLPs. A standard curve was constructed by coating the wells with serial dilutions of purified HA-histag proteins in mixture with 1 µg SIV Gag VLPs and was used for calculating the amount of chimeric proteins in VLPs.

After characterization of the HA/gp41 and HA-C14S/gp41 chimeric proteins, the genes for HA/gp41 and HA-C14S/gp41 were used to generate recombinant baculoviruses (rBV), designated as rBV-HA/gp41 and rBV-HA-C14S/gp41 respectively. These rBVs were then used to produce VLPs by coinfection of SF9 insect cells with rBV-SIV-Gag, a recombinant baculovirus that express the SIVmac239 Gag protein. Incorporation of the HA/gp41 or HA-C14S/gp41 chimeric proteins into VLPs were examined by immuno-gold labeling coupled with electron microscopy. As shown in Figure 2A, the HA/gp41 and HA-C14S/gp41 VLPs exhibit similar morphology and size to the SIV Gag VLPs, and both chimeric proteins were detected on the surface of VLPs by immuno-gold labeling while no gold-particle labeling was seen on SIV Gag VLPs. We further compared the oligomerization state of HA-C14S/gp41 and HA/gp41 chimeric proteins in VLP preparations by SDS-PAGE and Western blot analysis under non-reducing conditions. As shown in Figure 2B, while trimers of the chimeric proteins were detected in both HA-C14S/gp41 and HA/gp41 VLPs, HA-C14S/gp41 maintained a significantly higher portion of trimers than HA/gp41. Monomers were also detected in both HA-C14S/gp41 and HA/gp41 VLPs as expected since the protein oligomers are held by non-covalent bonds and can dissociate from each other in SDS-PAGE gels. We further compared the levels of HA/gp41 and HA-C14S/gp41 chimeric proteins incorporated in VLPs by ELISA. As shown in Figure 2C, similar amount of HA/gp41 or HA-C14S/gp41 chimeric proteins was detected in VLP preparations with approximately 20 ng chimeric protein in 1 µg VLPs, representing about 2% of the total proteins in VLP preparations.

### Immunization of guinea pigs with HA-C14S/gp41 DNA or VLP vaccines induces antibodies against the HIV gp41 that exhibit HIV neutralizing activity

**Figure 3:**
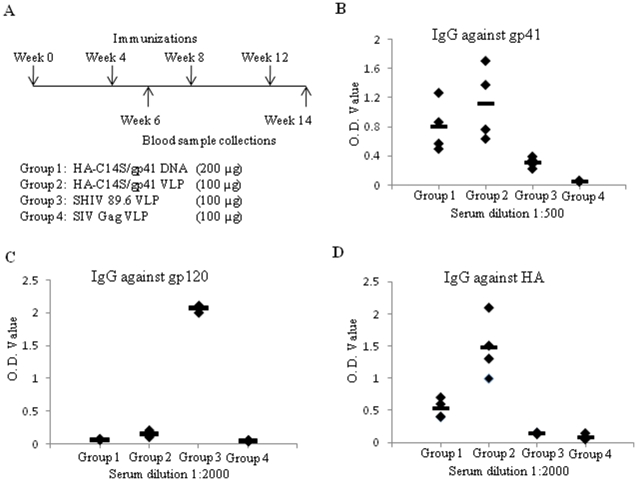
Immunization of guinea pigs and analysis of antibody responses. Guinea pigs (groups of 4) were immunized 4 times at 4-week intervals with HA-C14S/gp41 DNA (Group 1, 200 µg DNA per guinea pig per dose), HA-C14S/gp41 VLP (Group 2, 100 µg VLP per guinea pig per dose), SHIV 89.6 VLP (Group 4, 100 µg VLP per guinea pig per dose), or SIV Gag VLP (Group 4, 100 µg VLP per guinea pig per dose) as indicated, and blood samples were collected at 2 weeks after the second and fourth immunizations (**A**). The levels of antibody responses against HIV gp41 (**B**), HIV gp120 (**C**), or influenza HA (**D**) in sera from individual guinea pigs after 4 immunizations were determined by ELISA as described in Materials and Methods, and were expressed by the O.D. values at indicated serum dilutions for each antigen.

As shown in the above studies, the HA-C14S/gp41 chimeric protein resembles more closely to the wild type HA in conformation, exhibits improved immunogenicity in eliciting antibodies against gp41, and forms more stable trimers in VLPs. Based on these observations, the HA-C14S/gp41 DNA and VLP vaccines were selected for immunization of guinea pigs to evaluate their immunogenicities to induce HIV NAbs. As outlined in Figure 3A, four groups of guinea pigs (4 in each group) were used in this study and they were immunized 4 times by intramuscular injections at 4-week intervals. Group 1 received 200 µg HA-C14S/gp41 DNA vaccine and Group 2 received 100 µg HA-C14S/gp41 VLPs. A third group (Group 3) received 100 µg SHIV 89.6 VLPs, which are comprised of SIV Gag and HIV 89.6 Env (from which the gp41 portion of the HA-C14S/gp41 chimeric protein is derived). A fourth group (Group 4) that serves as a control group received 100 µg SIV Gag VLPs. Blood samples were collected at 2 weeks after the second and fourth immunizations for analysis of antibody responses. Only low levels of antibodies against gp41 were induced after 2 immunizations in Groups 1 and 2 that were immunized with HA-C14S/gp41 DNA or VLP vaccines (data not shown). After 4 immunizations, both HA-C14S/gp41 DNA and VLP vaccines induced significant levels of antibody responses against gp41 (Figure 3B). Although immunization with HA-C14S/gp41 VLPs induced higher levels of gp41-specific antibodies on average than those induced by HA-C14S/gp41 DNA vaccines, the differences are not statistically significant between these two groups (p>0.05). In contrast, only low levels of gp41-specific antibodies were induced by immunization with SHIV 89.6 VLPs, which were only slightly above the background levels as detected in sera from control group guinea pigs (Figure 3B). However, immunization with SHIV 89.6 VLPs induced high levels of antibody responses against the HIV gp120, whereas no significant level of gp120-specific antibodies was induced by HA-C14S/gp41 DNA or VLP vaccines (Figure 3C). In addition, immunization with both HA-C14S/gp41 DNA and VLP vaccines also induced antibody responses against the HA protein of the Aichi influenza virus, from which the HA1 subunit in the HA-C14S/gp41 chimeric protein is derived (Figure 3D). Of interest, while immunization with HA-C14S/gp41 DNA and VLP vaccines induced similar levels of gp41-specific antibodies, antibody responses against HA were significantly higher in guinea pigs immunized with HA-C14S/gp41 VLPs than those induced by the HA-C14S/gp41 DNA vaccine (p<0.05).

**Figure 4:**
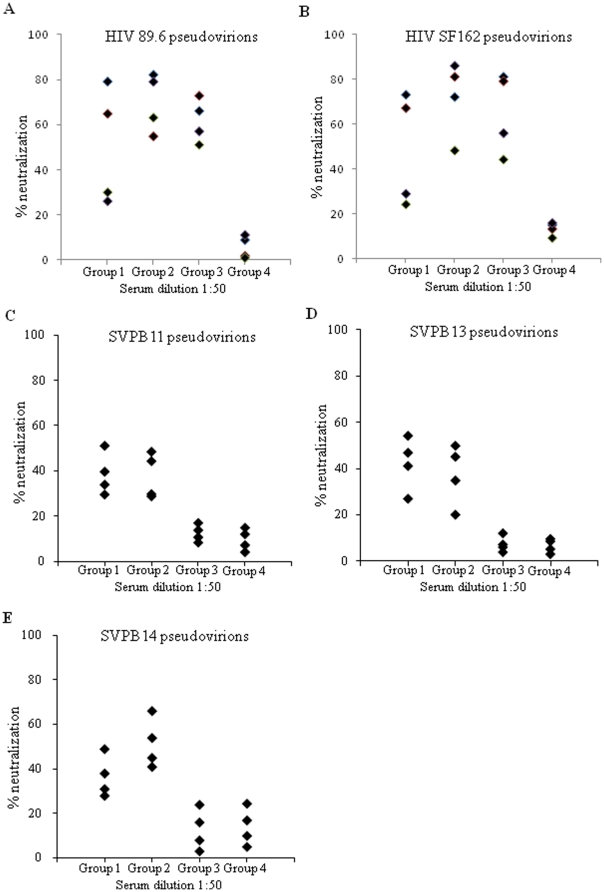
Neutralization of HIV pseudovirions. Guinea pigs were immunized as described in Figure 3A and sera collected at 2 weeks after the fourth immunization were analyzed for HIV neutralizing activity using a pseudovirion neutralization assay as described in Materials and Methods. HIV pseudovirions were incubated with individual serum samples from vaccinated guinea pigs at 1∶50 dilutions in 10% FCS-DMEM for 1 hr at 37°C and then added to TMZ-bl cells seeded in a 96-well plate. Two days after infection, the medium was removed and the cells were fixed and stained for β-galactosidase expressing cells. Neutralizing activity was expressed as the percentage of reduction in the number of β-galactosidase expressing cells in sample wells compared to those in control wells (% neutralization). Group 1, HA-C14S/gp41 DNA; Group 2, HA-C14S/gp41 VLP; Group 3, SHIV 89.6 VLP; Group 4, SIV Gag VLP. A. Neutralization of HIV 89.6 pseudovirions; B, Neutralization of HIV SF162 pseudovirions; C, Neutralization of HIV PVO.4 (SVPB11) pseudovirions; D, Neutralization of HIV AC10.029 (SVPB13) pseudovirions; E, Neutralization of HIV PRHPA4259.7 (SVPB14) pseudovirions.

To determine whether HIV NAbs were induced, we analyzed the activity of sera from immunized guinea pigs to neutralize HIV pseudovirions. As expected, immunization by SHIV 89.6 VLPs induced NAbs against HIV 89.6 as well as SF162 pseudovirions while no significant neutralizing activity was detected in sera from guinea pigs immunized with the control SIV Gag VLPs (Figure 4A and 4B). Further, sera from all guinea pigs immunized with HA-C14S/gp41 VLPs neutralized HIV 89.6 and SF162 pseudovirions at significant levels that are comparable to those induced by SHIV 89.6 VLPs. In comparison, sera from 2 out of 4 guinea pigs immunized with the HA-C14S/gp41 DNA vaccine neutralized over 50% HIV 89.6 and SF612 pseudovirions at 1∶50 dilution. In correlation with antibody responses against gp41, sera with low levels of gp41-specific antibodies neutralized HIV pseudovirions relatively poorly compared to sera with higher levels of gp41-specific antibodies. We further investigated whether immunization with HA-C14S/gp41 DNA and VLP vaccines induced HIV bNAbs by assessing their neutralizing activity against HIV pseudoviruses prepared with primary HIV Env proteins. Three tier 2 HIV Env including PVO.4 (SVPB11), AC10.029 (SVPB13), and PRHPA4259 (SVPB 14) were selected from the standard HIV subtype B panel compiled by Li et al. [36], which exhibit moderate or low neutralization-sensitivity to HIV-1-positive serum and high resistance to neutralization by V3-specific HIV antibodies. As shown in Figure 4 (C–E), sera from guinea pigs immunized with HA-C14S/gp41 DNA or VLP vaccines also neutralized these pseudoviruses, although at reduced levels compared to neutralization of HIV 89.6 and SF162 pseudovirions. In contrast, sera from guinea pigs immunized with SHIV 89.6 VLPs did not exhibit significant neutralizing activities against these pseudoviruses, similar to immunization with the control SIV Gag VLPs. These results show that immunization with HA-C14S/gp41 DNA and VLP vaccines induced NAbs against both tier 1 and tier 2 HIV pseudoviruses, whereas immunization with SHIV 89.6 VLPs only induced NAbs against tier 1 HIV pseudoviruses containing the homologous HIV 89.6 or the relatively easy-to-neutralize HIV SF162 Env proteins.

### Immunization with HA-C14S/gp41 DNA and VLP vaccines induced MPER-specific HIV NAbs

**Figure 5:**
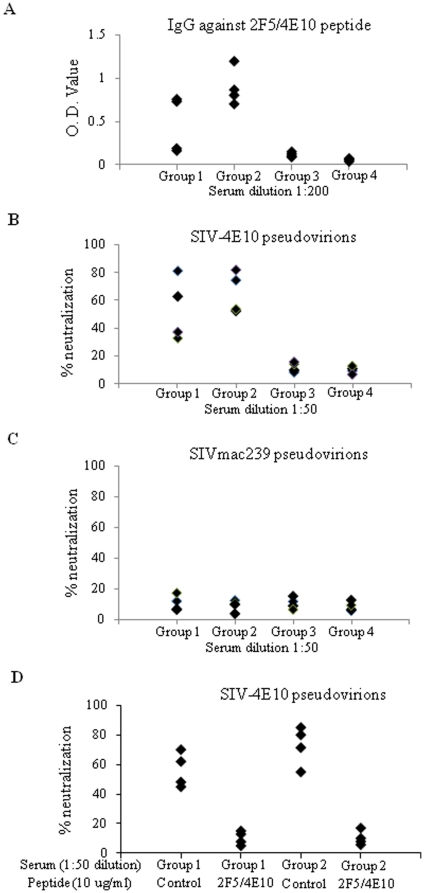
Induction of MPER-specific HIV NAbs by immunization with HA-C14S/gp41 DNA and VLP vaccines. Guinea pigs were immunized as described in Figure 3A and sera collected at 2 weeks after the fourth immunization were analyzed for the levels of MPER-specific antibody responses by ELISA as well as their neutralizing activity against a conserved epitope in MPER that was recognized by the monoclonal antibody 4E10 by a pseudovirion neutralization assay. **A.** Antibody responses against the MPER were determined by ELISA using the 2F5/4E10 peptide (ELLELDKWASLWNWFNITNW, a synthetic peptide corresponding to a segment in the MPER) as coating antigens, and expressed as the O.D. values for each serum sample at 1∶200 dilutions. SIV-4E10 (**B**) or SIVmac239 (**C**) pseudovirions were incubated with individual serum samples from vaccinated guinea pigs at 1∶50 dilutions in 10% FCS-DMEM for 1 hr at 37°C and then added to TZM-bl cells seeded in a 96-well plate. Two days after infection, the medium was removed and the cells were fixed and stained for β-galactosidase expressing cells. Neutralizing activity was expressed as the percentage of reduction in the number of β-galactosidase expressing cells in sample wells compared to those in control wells (% neutralization). **D.** Peptide competition of SIV-4E10 pseudovirus neutralization by sera from guinea pigs vaccinated by HA-C14S/gp41 DNA and VLP vaccines. Guinea pig sera (Group 1 and Group 2) were diluted in 10% FCS-DMEM at 1∶40 and pre-incubated with 0.1 ug of the 2F5/4E10 peptide (ELLELDKWASLWNWFNITNW) or a control peptide (AMQMLKETI, corresponding to a segment in the HIV Gag protein) at 37°C for 1 hr as indicated, and then used in neutralization assay (at a final dilution of 1∶50) against the SIV-4E10 pseudovirus. Group 1, HA-C14S/gp41 DNA; Group 2, HA-C14S/gp41 VLP.

To determine whether antibodies against conserved neutralizing epitopes in the MPER were induced, we compared the reactivity of guinea immune sera against a synthetic peptide corresponding to the epitopes recognized by MAbs 2F5 and 4E10. As shown in Figure 5A, HA-C14S/gp41 DNA or VLP vaccines induced higher levels of antibodies against the 2F5/4E10 peptide than the SHIV 89.6 VLPs. To investigate whether immunization with HA-C14S/gp41 DNA or VLP vaccines induced MPER-specific HIV NAbs, we constructed three chimeric proteins SIV-2F5, SIV-4E10, and SIV-2F5/4E10 by engrafting the core epitope for MAb 4E10, MAb 2F5, or both respectively into the SIVmac239 Env protein similarly as described by Yuste et al. [37]. In addition, we also introduced a truncation mutation in the cytoplasmic domain (leaving 17 amino acids in the cytoplasmic tail) which has been shown previously to increase surface expression and viral incorporation of the SIVmac239 Env [38]. Characterization of the SIV-4E10 chimeric protein showed that it was expressed on the cell surfaces, exhibited cell fusion activities, and was able to efficiently mediate pseudovirus infection. However, the SIV-2F5 and SIV-2F5/4E10 chimeric proteins exhibited low levels of cell fusion activity and a poor ability to mediate pseudovirus infection, similar as reported by Yuste et al. in their studies [37]. Further, the SIV-4E10 pseudoviruses were neutralized by monoclonal antibody 4E10 as well as by sera from SIVmac239 infected monkeys, but were resistant to neutralization by sera from HIV-infected patients (data not shown). We thus employed the SIV-4E10-pseudoviruses to test whether NAbs against the epitopes in the MPER of HIV gp41 were induced in guinea pigs immunized with HA-C14S/gp41 DNA or VLP vaccines. As shown in Figure 5B, sera from guinea pigs immunized with HA/gp41 DNA or VLP vaccines effectively neutralized SIV-4E10 pseudoviruses, at levels similar to the neutralization of HIV SF162 or 89.6 pseudoviruses. In contrast, sera from guinea pigs immunized with SHIV 89.6 VLPs exhibited only low neutralizing activity against SIV-4E10 pseudoviruses that was only slightly above the background levels as seen in sera from control guinea pigs. Further, none of the serum samples neutralized infection by SIVmac239-pseudoviruses (Figure 5C), indicating that NAbs induced by the HA-C14S/gp41 DNA and VLP vaccines are specific for the conserved epitopes in the MPER. To further confirm that neutralization of the SIV-4E10 pseudovirions was mediated by antibodies against epitopes in the MPER, we carried out a peptide competition assay. As shown in Figure 5D, pre-incubation of sera from guinea pigs vaccinated with HA-C14S/gp41 DNA or VLPs with the 2F5/4E10 peptide but not a control peptide effectively blocked their neutralizing activity against the SIV-4E10 pseudovirions. Taken together, these results further demonstrate that immunization with HA-C14S/gp41 DNA or VLPs induced MPER-specific HIV NAbs in guinea pigs.

## Discussion

Despite the sequence variation of the HIV Env protein, several conserved neutralizing epitopes have been identified through studies with human mAbs that exhibit broadly neutralizing activities against HIV [13], [15]. Among these conserved neutralizing epitopes, the MPER of gp41 has received great attention and widely recognized as a promising target for inducing HIV bNAbs [21], [27]. However, development of a vaccine strategy to induce HIV bNAbs against the conserved epitopes in MPER has been a difficult task. In this study, we investigated the ability of a chimeric protein HA/gp41, that exhibits enhanced reactivity to monoclonal antibodies 2F5 and 4E10, to induce HIV bNAbs in guinea pigs. Our results show that by mutating an unpaired Cys residue in its HA1 subunit to a Ser residue, the modified chimeric protein HA-C14S/gp41 exhibits increased reactivity to a conformation-sensitive monoclonal antibody against HA and forms more stable trimers in VLPs compared to the HA/gp41 chimeric protein. Further, immunization of guinea pigs using the HA-C14S/gp41 DNA and VLP vaccines induced antibodies against the HIV gp41 that exhibit broadly HIV neutralizing activities. Moreover, by using SIV-4E10 pseudovirions, we confirmed induction of HIV NAbs against the conserved epitopes in the MPER of HIV gp41 by immunization with the HA-C14S/gp41 DNA and VLP vaccines but not with SHIV 89.6 VLPs. Our results demonstrate for the first time the induction of MPER-specific HIV NAbs by a rationally designed vaccine strategy.

While the broadly neutralizing MAbs 2F5 and 4E10 against the MPER were obtained from an HIV-infected patient, the presence of such HIV NAbs is rare and can only be detected at low levels in a small portion of HIV-infected patients [37], [39]–[42]. One widely recognized difficulty for inducing such HIV NAbs is that the MPER is poorly accessible in native HIV Env due to shielding by the gp120 subunit [16], [21], [27], [43]. To overcome this difficulty, a number of strategies have been investigated to induce HIV NAbs against the conserved epitopes in the MPER including but not limited to: engrafting the 2F5 epitope core sequence ELDKWAS in the antigenic B site of an influenza virus HA [44]; construction of a fusion protein between the gp41 MPER segment and the porcine endogenous retrovirus Env p15E subunit [45]; grafting the gp41 MPER segment into the V1/V2 region of HIV-1 Env gp120 [46]; presenting gp41 derivatives in pre-fusion state on HIV VLPs [47]; presenting the MPER of gp41 on hepatitis B virus (HBV) surface antigen particles [32]; construction of epitope-scaffolds to present 2F5 and 4E10 epitopes [48], [49]; as well as by production of a trimeric MPER containing HA-gp41 fusion protein [33]. While these different vaccine strategies induced antibodies against the HIV gp41 and/or peptides corresponding to the epitopes in the MPER, little success was achieved in eliciting HIV NAbs. Thus, simply presenting the MPER segment in a more accessible form alone may not be sufficient to induce HIV NAbs. Structural analysis of the MAb 2F5 in complex with a peptide corresponding to its epitope showed that 2F5 only binds to one face of the peptide [50]. Further, the MPER has also been reported to form a kinked-helix conformation on the membrane surface that is partially buried into the membrane lipids [51]. Taken together, these results indicate that it may be necessary to present the MPER in a proper conformation to induce HIV NAbs in addition to enhancing the exposure of these epitopes.

The results presented in this study show that the induction of MPER-specific HIV NAbs can be achieved by a vaccine strategy that meets the requirements mentioned above. In the design of the HA/gp41 chimeric protein, the replacement of HIV gp120 with a relatively smaller HA1 subunit from influenza virus HA resulted in enhanced exposure of the MPER in gp41 and increased binding activity to monoclonal antibodies 2F5 and 4E10 [34]. In the present study, the HA/gp41 was further modified by mutation of an unpaired Cys residue in the HA1 subunit and the resulting HA-C14S/gp41 chimeric protein exhibits similar reactivity to MAbs 2F5 and 4E10 compared to HA/gp41, indicating that the exposure of the MPER in the chimeric protein is not affected by the mutation. Moreover, the HA-C14S/gp41 chimeric protein retained reactivity to a conformation sensitive MAb HC67 against the HA1 subunit, indicating that the folding of HA-C14S/gp41 resembles the wild type HA more closely in conformation. Further, the HA-C14S/gp41 chimeric protein is also found to form more stable trimers in VLP preparations. In addition, in both the DNA and VLP vaccine platforms used in this study, the HA-C14S/gp41 chimeric protein is presented to the immune system in its membrane anchored form. Thus, the key difference of this vaccine strategy as compared to the others is that the MPER in the HA-C14S/gp41 chimeric protein is presented in conformation that more closely mimics its natural state, which may play a critical role in the successful induction of MPER-specific HIV NAbs. By immunization studies in guinea pigs, it was observed that the HA-C14S/gp41 DNA and VLPs vaccines induced higher levels of antibodies against the HIV gp41 as well as a peptide corresponding to the MPER than SHIV 89.6 VLPs. Further, sera from HA-C14S/gp41 DNA- or VLP-vaccinated guinea pigs exhibited neutralizing activity against not only the HIV 89.6 and SF162 pseudoviruses but also four tier 2 primary HIV pseudoviruses as well as the chimeric SIV-4E10 pseudoviruses. In contrast, sera from SHIV VLP-vaccinated guinea pigs only neutralized HIV 89.6 and SF162 pseudoviruses but not the tier 2 primary HIV pseudoviruses or the SIV-4E10 pseudovruses. These results demonstrate that the HA-C14S/gp41 DNA and VLPs are more effective for eliciting MPER specific HIV bNAbs than the SHIV 89.6 VLPs that contain a wild type HIV Env protein.

While the induction of MPER-specific HIV bNAbs represents a significant advancement towards our goal, it is also noted that the levels of such antibodies are still low. The low level of neutralizing activity probably resulted from the relatively low level of MPER-specific antibody responses induce by the HA-C14S/gp41 DNA and VLP vaccines. This is similar to observations made using the HBV surface antigen as a carrier, which also presents the MPER in its membrane proximal form [32]. On the other hand, immunization with a purified HA-gp41 chimeric protein that consists the Heptad Repeat 1 (HR1) region of influenza HA plus the Heptad Repeat 2 (HR2) and MPER regions of gp41 induced high levels of antibodies against the HIV gp41, but no HIV NAbs were detected [33]. Taken together, these results indicate that the immunogenicity of the epitopes in the MPER may be weak when the MPER is presented in its membrane associated conformation. In addition, our results show that while immunization with HA-C14S/gp41 DNA and VLP vaccines induced similar levels of gp41 as well as MPER-specific antibodies, immunization with VLPs induced significantly higher levels of antibody responses against the HA1 subunit of the chimeric protein. This observation indicates that inducing an antibody response to weakly immunogenic epitopes may not be effectively augmented simply by using a more immunogenic vaccine platform. Further, while immunization with SHIV 89.6 VLPs also induced low levels of antibodies against gp41 as well as the 2F5/4E10 peptide, no significant neutralization of the SIV-4E10 pseudovirions was observed. Thus, it is also possible that not all MPER-specific antibodies induced by vaccinations are capable of neutralizing HIV infection. Additional studies are necessary to determine the properties of the MPER-specific HIV NAbs and to develop strategies to boost induction of such antibodies.

In summary, the results in this study show that HIV NAbs can be successfully induced against the conserved epitopes in the HIV Env MPER. However, the neutralizing activity induced by HA-C14S/gp41 DNA and VLP vaccines against HIV pseudovirions, especially those containing Env from primary HIV isolates, is still relatively weak. Further, the level of MPER-binding antibodies induced by HA/gp41 based vaccines seems also to be lower compared to other studies that reported induction of high levels of MPER-binding antibodies with little to no HIV neutralizing activity[33], [46], [47]. One possible explanation for these seemingly paradoxical observations is that induction of such antibodies may require presentation of MPER in a proper membrane associated conformation and the MPER presented in such a form only exhibits weak immunogenicity. Thus, new strategies are still needed to be investigated to overcome this difficult and yet important issue. Nonetheless, the success of this study paves the way for future studies to development a more effective vaccine strategy to induce HIV bNAbs against the conserved epitopes in the MPER of the HIV Env.

## Materials and Methods

### Ethics statement

Animal ethics approval for the immunization studies in mice and guinea pigs was obtained from the Institutional Animal Care and Use Committee (IACUC) at the Emory University. All animal studies were performed in compliance with the guidelines of the Institutional Animal Care and Use Committee (IACUC) under approval from the IACUC at the Emory University.

### Cells, reagents, and animals

Hela cells and 293T cells were obtained from American Type Culture Collection (ATCC) and TMZ-bl cells were obtained from NIH AIDS Research and Reference Reagent Program (NIH ARRRP). The cells were maintained in DMEM supplemented with 10% fetal calf serum (FCS-DMEM). Human MAbs 2F5 and 4E10 and polyclonal antibodies against HIV (HIVIG) were obtained from NIH ARRRP. Rabbit polyclonal antibodies against the HA of influenza virus Aichi (Jose 4) and mouse MAb HC-67 that recognizes a conformational sensitive epitope in HA [35] were kindly provided by Dr. David Steinhauer. Female Balb/c mice (8-week old) and female guinea pigs (2 month old, weight 250 g) were purchased from the Jackson Laboratory and housed in the animal facility at the Emory University.

### Gene construction and analysis of protein expression

Construction of the HA/gp41 gene has been described previously ([34]). For construction of the HA-C14S/gp41 gene, the codon for the Cys 14 residue in the HA/gp41 gene was changed into the codon for a Ser residue by site-directed mutagenesis (amino acid sequence of the HA-C14S chimeric protein were provided in Figure S1). Protein expression was analyzed using the recombinant vaccinia virus T7 (VTF7-3) transient expression system. Briefly, 10×6 HeLa cells were seeded in 6-well plate and grown to 90% confluency overnight. The cells were then infected with VTF7-3 which expresses the T7 polymerase for 2 hr followed by transfection with indicated DNA constructs using Lipofectin (Invitrogen). At 12 hr post transfection, cells were starved in Met, Cys-deficient DMEM for 30 min and then labeled with 100 uCi ^35^S-Met,Cys labeling mix (Amersham) in 1 ml Met, Cys-deficient DMEM for 4 hr. Following radioactive labeling, protein surface expression was detected by a surface biotinylation assay as described in our previous studies [52], [53]. Briefly, after labeling, cells were washed with phosphate-buffered saline (PBS) at 4°C and then incubated with 2 ml of NHS-SS-biotin dissolved in PBS (0.5 mg/ml) for 30 min at 4°C. The cells were then lysed with lysis buffer (150 mM NaCl/50 mM Tris HCl, pH 7.5/1 mM ethylenediamine tetraacetate/1% Triton X-100/1% sodium deoxycholate), and then immunoprecipitated with Jose4 antibodies and Protein A-agarose bead at 4°C overnight. After immunoprecipitation, samples were washed twice in lysis buffer plus 0.4% SDS and then split into two equal portions. One portion (representing total cellular expression of the chimeric proteins) was mixed with 15 ul sample buffer (125 mM Tris-HCl [pH 7.5], 4% SDS, 20% glycerol, plus 10% ß-mercaptoethanol) and heated at 95°C for 5 min before being analyzed by SDS-PAGE. The other portion was mixed with 20 ul 10% SDS and heated at 95°C for 15 min to dissociate protein-antibody-Protein A-agarose bead complex. Dissociated proteins were then dissolved in 700 ul lysis buffer and incubated with 10 ul Streptavidin-agarose bead for 5 h at 4°C to precipitate biotinylated surface proteins. Biotinylated samples (representing surface expression of the chimeric proteins) were washed three times with lysis buffer, mixed with 15 ul sample buffer, and heated at 95°C for 5 min before analysis by SDS-PAGE.

Inparing reactivity of the chimeric proteins expressed on the cell surface with different antibodies, we employed a cell surface ELISA following established protocols [34]. Briefly, protein expression was carried out in HeLa cells using the VTF7-3 transient expression system. At 16 hr post transfection, the cells were fixed with 0.5% glutaradehyde in PBS and then blocked with PBS containing 5% (w/v) BSA. After washing with PBS, the cells were incubated with the indicated primary antibodies (2F5, 4E10, HC67, or Jose 4 diluted in PBS at 1∶1000) for 2 hr at room temperature, washed again with PBS, and then incubated with corresponding HRP-conjugated secondary antibodies diluted at 1∶4000 in PBS (Goat-anti-human for 2F5 and 4E10, Goat-anti-mouse for HC67, and Goat-anti-rabbit for Jose4 antibodies respectively, R&D Systems) for 1 hr at room temperature. The cell were washed again with PBS to remove unbound antibodies and then substrate TMB (3,3′,5,5′-tetramethylbenzidine, Sigma) was added for development of color. The O.D. values, which represent the relative amount of bound antibody, were read with an ELISA plate reader at the wavelength of 450 nm.

### VLP production and characterization

VLPs were produced in Sf9 insect cells (Invitrogen) using the recombinant baculovirus (rBVs) expression system. Construction of rBVs expressing the SIV Gag and HIV 89.6 Env proteins and production of SHIV 89.6 VLPs have been described previously [54], [55]. The genes for the HA/gp41 and HA-C14S/gp41chiemric proteins were cloned into the plasmid vector pFastBac (Invitrogen) under the Pphol promoter and rBVs were generated using the Bac-to-Bac Bacmid system (Invitrogen) following the manufacturer's protocol, which were designated as rBV-HA/gp41 and rBV-HA-C14S/gp41 respectively. For VLP production, Sf9 cells (2×10^6^/ml) were co-infected by rBV-SIV Gag along with rBV-HA/gp41 or rBV-HA-C14S/gp41 at MOIs (multiplicity of infection) of 2 and 5 respectively, and VLPs released into the medium were collected at 60 hr post infection. After clarification of cell debris, VLPs were concentrated by ultra-centrifugation at 28,000 rpm for 40 min in an SW-28 rotor (Beckman)and further purified through a discontinuous sucrose gradient (10–50%) by ultra-centrifugation at 32,000 rpm for 1 hr in an SW-41 rotor (Beckman). Purified VLPs were then concentrated by ultra-centrifugation at 28,000 rpm for 40 min in an SW-28 rotor (Beckman) and re-suspended in PBS. Protein concentration of VLPs was determined using a BCA assay kit and the VLP preparations were adjusted with PBS to a final protein concentration of 1 mg/ml. SIV Gag VLPs, which were used as control VLPs throughout this study, were produced by infecting Sf9 cells with rBV-SIV-Gag alone and similarly purified through a sucrose gradient.

Purified VLPs were characterized by Western blot for the presence of HA/gp41 or HA-C14S/gp41 chiemric proteins. For detecting oligomeric forms of HA/gp41 or HA-C14S/gp41 chiemric proteins in VLPs, samples were prepared by addition of non-reducing sample buffer and heated at 72^o^C for 5 min prior to analysis by SDS-PAGE followed by Western blot using rabbit-anti-HA antibodies (Jose4, diluted at 1∶4000 in PBS) as the primary antibody and HRP-conjugated, goat-anti-rabbit-IgG antibodies as the secondary antibody (diluted at 1∶5000 in PBS). Incorporation of the HA/gp41 or HA-C14S/gp41 chimeric proteins in VLPs was further examined by immunogold-particle labeling and electron microscopy (EM). Briefly, purified VLPs were coated onto a sample grid and incubated with rabbit-anti-HA antibodies for 1 hr at room temperature. After removing unbound antibodies by rinsing in PBS, the samples were incubated with gold-particle-conjugated goat-anti-rabbit secondary antibodies for 1 hr at room temperature, washed again by rinsing in PBS, followed by negative staining with 1% (w/v) uranyl acetate and then examined with a Hitachi-H7500 transmission electron microscope. The amounts of HA/gp41 and HA-C14S/gp41 chimeric proteins in VLP preparations were further determined by ELISA. VLPs were lysed with 1% Triton X-100 and then coated onto a microtiter plate at 1 µg per well in triplicates. Chimeric proteins were detected by incubation with rabbit-anti-HA antibody (Jose 4) as the primary antibody and then HRP-conjugated, goat-anti-rabbit-IgG antibodies as the secondary antibody. Control wells were coated with 1 µg of SIV-Gag VLPs. After a final wash, ABTS (2,2′-azino-bis(3-ethybenz-thiazoline-6-sulfonic acid), Sigma) dissolved in citrate phosphate buffer (3 mg ABTS in 10 ml CPB, pH 4.2, plus 10 µl H_2_O_2_) was added at 100 µl/well to develop color and read by an ELISA plate reader at 405 nm. Absorption values (O.D values) were recorded and presented as the levels of chuimeric proteins in VLPs for comparison. A standard curve was constructed by coating the microtiter plate with serial dilutions of purified HA-histag protein mixed with 1 µg SIV Gag VLPs for calculating the amount of chimeric proteins in VLPs.

### Immunizations

The genes for the HA/gp41 and HA-C14S/gp41 chimeric proteins were cloned into the plasmid vector pCAGGS as described previously [34], [52], [53]. Plasmids were amplified in E. coli DH5α, purified with a Qiagen Endo-Free Megaprep kit and dissolved in sterile PBS at 1 µg/ µl. Mice (groups of 6) were immunized twice at 4-week intervals with 100 µg of the indicated DNA construct by intra-muscular injection in both quadriceps, and blood samples were collected by retro-orbital bleeding at two weeks after the second immunization. Sera were heat-inactivated at 56°C for 30 min, and then stored at −80°C until analysis. Guinea pigs (groups of 4) were immunized four times at 4-week intervals with indicated the DNA vaccines (200 µg per guinea pig per immunization) or VLPs (100 µg per guinea pig per immunization) by intra-muscular injection in both quadriceps, and blood samples were collected at two weeks after the second and fourth immunizations through the cranial vena cava. Sera were heat-inactivated at 56°C for 30 min, and then stored at −80°C until analysis.

### Analysis of serum antibody response by ELISA

Serum samples were analyzed by ELISA for antibody responses against HIV gp41, HIV gp120, influenza virus Aichi HA, and a peptide corresponding to a segment in the MPER of gp41. Purified gp120-histag, HA-histag, and HN/gp41-histag proteins were produced as described previously [34], [52], [56]. Briefly, a recombinant vaccinia virus expressing a gp12-histag, HA-histag, or HN/gp41-histag protein (the gp120, HA1 subunit of Aichi influenza virus HA, or the HN-gp41 fusion protein ectodomain was fused with a histag segment HHHHHH at its C-terminus respectively) was generated and used to infect Hela cells. At 48 hr post-infection, histag proteins expressed in cells were purified using Ni-NTA agarose beads (QIAGEN, Valencia, CA) and then characterized by Coomassie Blue staining and Western blot. A peptide (ELLELDKWASLWNWFNITNW) corresponding to a segment of the gp41 MPER was synthesized at the Emory University Microchamical Facility, which contains the core epitopes for monoclonal antibodies 2F5 and 4E10 and was designated as the 2F5/4E10 peptide (>70% pure).

For analysis of antibody responses against these different antigens, microtiter plates were coated with different purified proteins or the 2F5/4E10 peptide at 200 ng/well overnight, and then incubated with collected serum samples diluted in PBS at different dilutions as indicated for 2 hr at room temperature. After wash, HRP-conjugated secondary antibodies (Sigma) against mouse or guinea pig IgG were added to the plate and incubated for 2 hr at room temperature. After a final wash, ABTS (2,2′-azino-bis(3-ethybenz-thiazoline-6-sulfonic acid), Sigma) dissolved in citrate phosphate buffer (3 mg ABTS in 10 ml CPB, pH 4.2, plus 10 µl H_2_O_2_) was added at 100 µl/well for developing color and read by an ELISA reader at 405 nm. Absorption values (O.D values) were recorded and presented as the levels of antigen specific antibodies for comparison.

### Analysis of serum neutralizing activity

Serum neutralizing activity was analyzed by a pseudovirion neutralization assay as described previously [34]. Pseudoviruses were produced by co-transfection of 293T cells with a cDNA construct for Env-deficient HIV NL-4 and a DNA construct expressing the HIV SF162, HIV 89.6, HIV PVO.4 (SVPB11), HIV AC10.029 (SVPB13), HIV PRHPA4259 (SVPB14), SIV-4E10, or SIVmac239 EnvTr proteins. The env genes for HIV SF162, HIV 89.6, HIV PVO.4 (SVPB11), HIV AC10.029 (SVPB13), and HIV PRHPA4259 (SVPB14) were obtained from NIH ARRRP. Construction of the SIVmac239 EnvTr has been described previously [38]. Construction of the SIV-4E10 env gene was carried out essentially as described by Yuste et al. [37], in which two amino acid residues in the SIVmac239 EnvTr, Leu-684 and Ala-685, were changed into the corresponding amino acid residues in the HIV 89.6 Env (Ile and Thr respectively) by site-directed mutagenesis. We also constructed SIV-2F5 and SIV-2F5/4E10 env genes by mutating amino acids Lys-671, Asn-673, Ser-674, Asp-676, and Val-677 in the backbone of SIVmac239 EnvTr or SIV-4E10 respectively following the same approach. However, SIV-2F5 and SIV-2F5/4E10 pseudovirions exhibited very low infectivity similarly as reported by Yuste et al. [37] and they were not used in neutralization assays. After preparation, pseudoviruses were tittered in TMZ-bl cells and stored at −80°C until use.

For analysis of sera neutralizing activity, TMZ-bl cells were seeded at 40,000 cells per well in a 96-well plate in 10% FCS-DMEM media overnight at 37°C with 5% CO_2_. Serum samples collected from guinea pigs that were heat-inactivated at 56°C for 30 min. Pseudoviruses (100 infectious particles) were incubated with heat-inactivated sera at 1∶50 dilution in 50 µl 10% FCS-DMEM. Pseudovirions incubated with 10% FCS-DMEM only were used as controls. The virus-serum mixtures were prepared in triplicates and incubated at 37°C for 1 hr and then added to TMZ-bl cells with an additional 150 µl of 10% FCS-DMEM plus DEAE-Dextran (at a final concentration of 15 µg/ml). Two days after infection, medium was removed and the cells were fixed and stained as described in our previous studies [57]. Neutralization was calculated as: (average number of blue foci in control wells – average number of blue foci in sample wells)/(average number of blue foci in control wells) X 100%, and presented as percentage of neutralization. Peptide competition was carried out to confirm MPER-specific neutralizing activity of sera from vaccinated guinea pigs. Briefly, 1 ul guinea pig sera were incubated with 0.1 ug of the 2F5/4E10 peptide or a control peptide (AMQMLKETI, which corresponds to a segment in the HIV Gag protein and was synthesized at the Emory University Microchemical Facility, purity>70%) in 40 ul 10% FCS-DMEM at 37°C for 1 hr, followed by addition of 100 infectious units of pseudoviruses diluted in 10 ul 10% FCS-DMEM (to a total volume of 50 ul and a final serum dilution at 1∶50) and further incubation at 37°C for 1 hr. After incubation, the peptide-serum-pseudovirus mixtures (prepared in triplicates) were added to TMZ-bl cells with an additional 150 µl of 10% FCS-DMEM plus DEAE-Dextran (at a final concentration of 15 µg/ml) to determine virus neutralization as described above.

## Supporting Information

Figure S1 Amino acid sequence for HA-CI4S/gp41. The Cys14-Ser mutation in the HA1 subunit indicated by (*) and amino acid sequence from HIV 89.6 Env gp41 was underlined.
(0.07 MB TIF)
