# Supplementary figures and images for: Induction of HIV Neutralizing Antibodies against the MPER of the HIV Envelope Protein by HA/gp41 Chimeric Protein-Based DNA and VLP Vaccines
Source: PLoS One. 2011 May 19;6(5):e14813. doi: 10.1371/journal.pone.0014813 (PMC3098228; doi:10.1371/journal.pone.0014813)

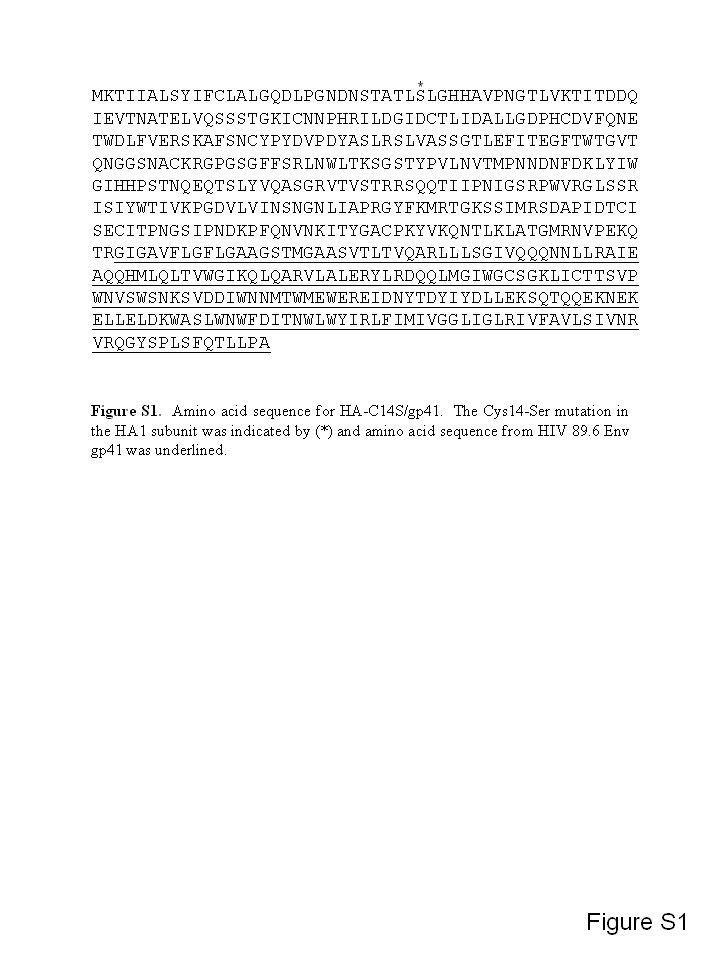

Supplement: Figure S1 — Amino acid sequence for HA-CI4S/gp41. The Cys14-Ser mutation in the HA1 subunit indicated by (*) and amino acid sequence from HIV 89.6 Env gp41 was underlined. (0.07 MB TIF) [file pone.0014813.s001.tif]
